# Resistance of *Listeria monocytogenes* to Stress Conditions Encountered in Food and Food Processing Environments

**DOI:** 10.3389/fmicb.2018.02700

**Published:** 2018-11-13

**Authors:** Florentina Ionela Bucur, Leontina Grigore-Gurgu, Peter Crauwels, Christian U. Riedel, Anca Ioana Nicolau

**Affiliations:** ^1^Faculty of Food Science and Engineering, Dunarea de Jos University of Galati, Galati, Romania; ^2^Institute of Microbiology and Biotechnology, Ulm University, Ulm, Germany

**Keywords:** acidity, temperature, oxidative stress, osmolarity, high pressure, UV light, pulsed electric fields, bacteriocins

## Abstract

*Listeria monocytogenes* is a human food-borne facultative intracellular pathogen that is resistant to a wide range of stress conditions. As a consequence, *L. monocytogenes* is extremely difficult to control along the entire food chain from production to storage and consumption. Frequent and recent outbreaks of *L. monocytogene*s infections illustrate that current measures of decontamination and preservation are suboptimal to control *L. monocytogenes* in food. In order to develop efficient measures to prevent contamination during processing and control growth during storage of food it is crucial to understand the mechanisms utilized by *L. monocytogenes* to tolerate the stress conditions in food matrices and food processing environments. Food-related stress conditions encountered by *L. monocytogenes* along the food chain are acidity, oxidative and osmotic stress, low or high temperatures, presence of bacteriocins and other preserving additives, and stresses as a consequence of applying alternative decontamination and preservation technologies such high hydrostatic pressure, pulsed and continuous UV light, pulsed electric fields (PEF). This review is aimed at providing a summary of the current knowledge on the response of *L. monocytogenes* toward these stresses and the mechanisms of stress resistance employed by this important food-borne bacterium. Circumstances when *L. monocytogenes* cells become more sensitive or more resistant are mentioned and existence of a cross-resistance when multiple stresses are present is pointed out.

## Introduction

Along the food chain, bacteria are constantly exposed to a wide range of stress factors, which affect their activity and viability. These stresses are either intrinsic to the food matrix or extrinsic factors intentionally applied to preserve food or imposed onto the organisms upon consumption by the host (Ruiz et al., 2017). *L. monocytogenes* is an important food-borne pathogen (Schlech et al., 1983) that frequently causes food recalls^1^^,^^2^ and disease outbreaks with significant case numbers and a mortality rate of 20–30%^3^ worldwide (Buchanan et al., 2017). This organism is known for its ability to survive or even to replicate under a wide range of environmental stress conditions (Gandhi and Chikindas, 2007; Ferreira et al., 2014; Gahan and Hill, 2014). Resistance to stress supports colonization and persistence of *L. monocytogenes* in various niches along the food chain and thus formation of reservoirs for contamination (Berrang et al., 2010; Leong et al., 2014, 2015; Bolocan et al., 2016). Moreover, it ultimately contributes to the ability of this bacterium to infect humans (Sleator et al., 2009).

The stresses encountered by *L. monocytogenes* in foods include those that are a consequence of various methods of preservation, including traditional ones as acidic pH due to fermentation by e.g., lactic acid bacteria (LAB), and osmotic stress by increased salt concentrations and more contemporary ones as using of growth inhibitors including bacteriocins and other food preservatives (Leroy and de Vuyst, 2004; Albarracín et al., 2011; Johnson et al., 2017). It should be mentioned that bacteriocins, which are small antimicrobial peptides, may either be naturally produced by bacteria used for food fermentation or can be added exogenously as a preserving additive. On the other hand, there are measures of food preservation that are rather technical in nature and are designed either to kill pathogens and spoilage microorganism at the processing stage [thermal treatments and its alternatives high pressure, pulsed electric fields (PEF), radiation] or even to protect foods during their storage (low temperatures/refrigeration, low oxygen concentrations, presence of protective gases in the surrounding atmosphere) (Morris et al., 2007; Rascon Escajeda et al., 2018).

The same stress may occur on several occasions along the food chain. For example, *L. monocytogenes* may be exposed to pH and osmotic stress first in the food matrix as consequence of fermentation or food preservation and subsequently in the host gastrointestinal tract. In this respect, it is of importance that resistance to different stresses is interconnected. For example incubation of *L. monocytogenes* at low temperatures enhances its resistance to high salt concentrations (Schmid et al., 2009). Likewise, osmotic stress in *L. monocytogenes* can lead to cross-protection against other causes of injury, including heat, ethanol, acidity, alkalinity, and oxidative stress (Melo et al., 2015). This is, at least partially explained by the fact that the stress signal received by the two component systems *liaRS*, *lisRK*, *cesRK*, *agrCA*, and *virRS*, which have been demonstrated to play a role in the stress response (Kang et al., 2015; Pöntinen et al., 2015, 2017), converge on the level of SigB, which is the alternative sigma factor σ^B^ that controls the general stress response in *L. monocytogenes* and other Gram-positive bacteria (Kazmierczak et al., 2003; Chaturongakul and Boor, 2006; Abram et al., 2008). For *L. monocytogenes*, SigB has been shown to be involved in the resistance to acidity (Wemekamp-Kamphuis et al., 2004b), osmotic stress (Fraser et al., 2003), cold and freezing stress (Becker et al., 2000), oxidative stress (Chaturongakul and Boor, 2004), and high hydrostatic pressure (Wemekamp-Kamphuis et al., 2004b). Appropriate resistance mechanisms are triggered by activation of σ^B^-dependent promoters (Van Schaik and Abee, 2005). The extremely high tolerance to stressful conditions makes *L. monocytogenes* a major concern in food processing and a suitable model organism to study resistance mechanisms to stress conditions encountered in food and food processing environments.

Some of the mechanisms (and consequences) of resistance of *L. monocytogenes* have been expertly reviewed previously (Doyle et al., 2001; Tasara and Stephan, 2006; Thévenot et al., 2006; Gandhi and Chikindas, 2007; Lungu et al., 2009; NicAogáin and O’Byrne, 2016). However, most of these reviews focus on osmotic, pH, and temperature stress. With the present review we aim at providing a summary of the current knowledge on resistance and associated mechanisms of *L. monocytogenes* with a clear focus on stressful conditions that arise from traditional or alternative methods of food processing, preservation and decontamination. While we will touch upon stresses reviewed elsewhere (acidic pH, osmolarity, high and low temperatures, oxidative stress), we will also discuss resistance of *L. monocytogenes* to other stress conditions that have not gained as much attention (bacteriocins, pulsed or continuous UV radiation or visible light, electrical fields, high pressure). A deeper understanding of the mechanisms used by *L. monocytogenes* to survive and proliferate in food products may help food specialists to design efficient preservation methods that will extend shelf lives and provide a better protection of consumers against this pathogen while at the same time maintain the sensory and nutritional properties of the food products.

## Resistance of *L. monocytogenes* to Stress During Food Processing and Storage

### Resistance to Thermal Stress

Thermal treatments and temperature control are strategies that have been applied in food production and preservation for centuries to prevent or limit contamination and outgrowth of food-borne pathogens. However, the efficacy of thermal treatments against *L. monocytogenes* is limited by the intrinsic ability of this pathogen to survive and actively replicate at temperatures between −0.4 and 45°C (Chaturongakul et al., 2008; Chan and Wiedmann, 2009).

#### Resistance to Thermal Treatments

Mild thermal treatments (<100°C) are largely applied in food processing in order to inactivate vegetative microbial cells of food-spoilage bacteria and food-borne pathogens. Such treatments ensure food safety and prolonged shelf life as long as food products are properly packed and adequately stored (Van Boekel et al., 2010). Despite these benefits, thermal treatments can have a negative impact on the quality of food affecting the nutritional value and sensory properties (Hardy et al., 1999). However, the main concern with thermal processing of foods remains the ability of sublethally injured pathogenic bacteria to recover and grow during post-processing storage. This is of particular relevance for *L. monocytogenes* with its ability to grow in a wide temperature range (Sörqvist, 1993; Mackey et al., 1994). Although *L. monocytogenes* does not manifest an extraordinary resistance to high temperatures, it was shown to be more heat tolerant than other non-spore-forming pathogens such as *Salmonella* and *E. coli* (Abdel Karem and Mattar, 2001; Huang, 2004; Sallami et al., 2006). Factors that influence the resistance of *L. monocytogenes* to heat vary among strains, bacterial cells’ age, test and growth conditions, previous environmental stresses, or food components (Doyle et al., 2001).

*L. monocytogenes* has been shown to survive the minimum high-temperature, short-time treatment imposed by U.S. Food and Drug Administration (71.7°C, 15 s) in the case of milk collected from deliberately contaminated cows. Early studies raised the possibility that polymorphonuclear leukocytes present in milk may have a protective effect on *L. monocytogenes* during heat treatments residing inside these cells (Fleming et al., 1985; Doyle et al., 1987). However, subsequent reports showed that, in naturally contaminated milk, *L. monocytogenes* was not able to resist temperatures greater than 67.5°C combined with a holding time of 16.2 s (Farber et al., 1988). Nevertheless, the conditions associated with dairy products seem to influence the resistance of *L. monocytogenes* to heat treatments. For instance, Casadei and colleagues showed that limited access to essential nutrients in butter and the physical structure of this food could induce a starvation state in *L. monocytogenes* cells correlated with cross-resistance to other types of stress. In this case *L. monocytogenes* Scott A grown within this food matrix was four times more resistant to a treatment at 60°C than the same strain grown in TSB broth (Casadei et al., 1998). Furthermore, *L. monocytogenes* was shown to survive relatively high temperatures in heat-treated meat (Farber et al., 1989; Gaze et al., 1989; Murphy et al., 2003), egg products (Bartlett and Hawke, 1995; Monfort et al., 2012) and vegetables such as mushrooms and peas (Mazzotta, 2001a).

Resistance of *L. monocytogenes* strains to heat can vary significantly among serotypes (Sörqvist, 1994). In one study, strains belonging to serotype 1/2a showed relatively low tolerance to heat (up to 2 log CFU/mL), while strains representing serotypes 1/2b and 4b exhibited an extensive variability (from undetectable to 4 log CFU/mL). The highest heat tolerance was recorded for a serotype 7 strain (5 log CFU/mL) (Shen et al., 2014).

*L. monocytogenes* cells exposed to sublethal stresses prior to thermal challenge can become considerably more heat resistant. Shen and colleagues found that exposure of *L. monocytogenes* to a temperature of 48°C for 30 min led to heat stress adaptation among bacterial cells. Moreover, subjection to this mild stress for a short period of time did not affect the capacity of growing (Shen et al., 2014). Salt was also shown to potentiate the ability of *L. monocytogenes* to withstand thermal treatments (Jørgensen et al., 1995). For instance, the D_63°C_ value, which is the time required to kill 90% of bacteria when exposed to the temperature of 63°C, for Scott A strain inoculated in egg products with 10% NaCl increased approximately 6 times in comparison with that of the same strain processed in egg products without salt (Bartlett and Hawke, 1995). This may be due to the protective effect of decreased water activity in the growth medium (Shebuski et al., 2000). Acidity is another factor that can influence bacteria’s thermotolerance. Acid-adaptation in fruit juices was shown to substantially increase the resistance of *L. monocytogenes* to subsequent heat treatment (Mazzotta, 2001b). Also, *L. monocytogenes* displays an increased heat tolerance and a significantly increased D_60°C_ value (2.2 min) in stationary compared to exponential growth phase (0.6 min) (Jørgensen et al., 1999).

**FIGURE 1:**
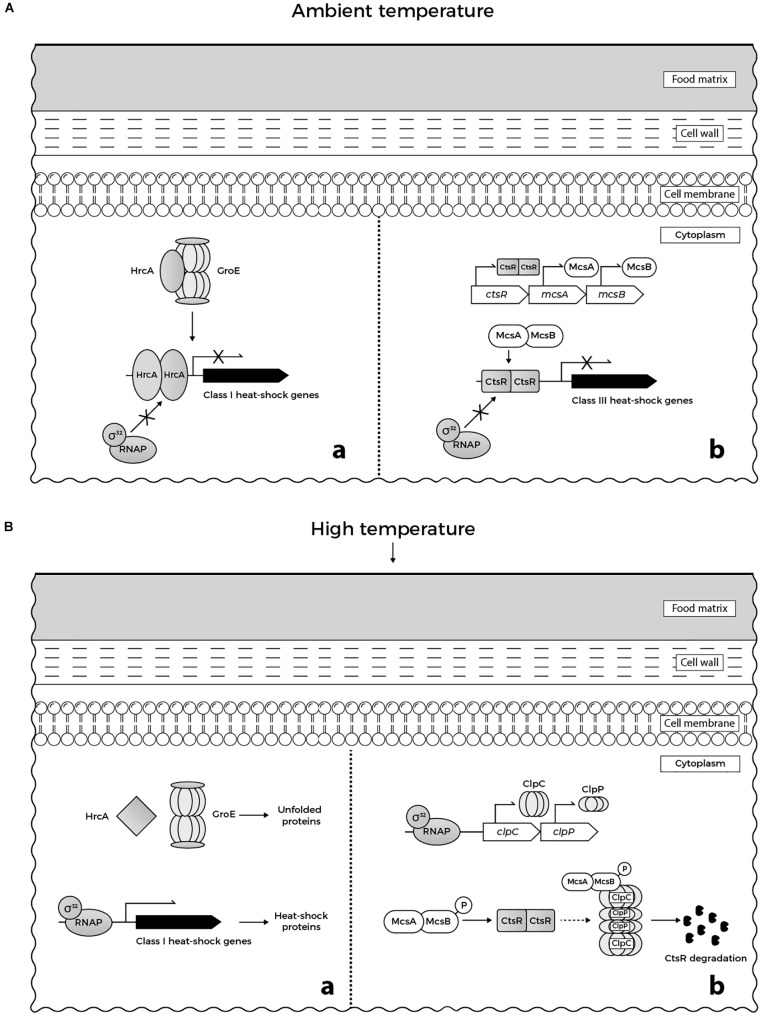
**(A)** Regulation of heat-shock genes in *L. monocytogenes* under ambient temperature in food matrices: **(a)** GroE chaperonine ensures the adequate folding of HrcA repressor. When folded correctly, the repressor binds to its target promoters, preventing expression of class I heat-shock genes. **(b)** McsB (tyrosine kinase) and its co-activator McsA (zinger finger protein) are involved in the regulation of CtsR repressor activity. CtsR, stabilized by McsA, binds to its target promoters, preventing expression of class III heat-shock genes. **(B)** Regulation of heat-shock genes in *L. monocytogenes* under heat stress in food matrices: **(a)** GroE is titrated by unfolded proteins that accumulate in cytoplasm and cannot interact with HrcA. When denatured upon heat stress, misfolded HrcA is unable to bind to the target DNA. Consequently, RNA polymerase-σ^32^ binds to the target promoters allowing the transcription of class I heat shock genes. **(b)** Similarly, CtsR undergoes heat-induced conformational changes that prevent its interaction with the target promoters. This allows binding of RNA polymerase-σ^32^ to the promoters of *clp* genes inducing the transcription of *clpC* and *clpP*. Following temperature-dependent autophosphorylation, McsB, assisted by McsA, targets CtsR to degradation by ClpCP protease (based on Krüger et al., 2001; Roncarati and Scarlato, 2017; Roncarati and Scarlato, 2018).

At the molecular level, the response of *L. monocytogenes* to 48°C involves the expression of genes belonging to specific heat-shock regulons, namely class I and class III heat-shock genes, and genes of the SigB-dependent class II stress response. The upregulation of *recA* expression, an activator of SOS response implicated in DNA repair could be also observed (Van der Veen et al., 2007). Class I heat-shock genes (*grpE*, *dnaK*, *dnaJ*, *groEL*, and *groES*) encode for heat-shock proteins (HSPs) that act as intra-cellular chaperones whose expression is increased when denatured proteins accumulate in cytoplasm (Figure 1Ba). The role of HSPs is to stabilize and assemble partially unfolded proteins, preventing their aggregation under stress conditions. Under physiological growth conditions at ambient temperature, expression of class I heat-shock genes is controlled by the HrcA repressor (Figure 1Aa), which in turn is encoded by the first gene of the *dnaK* operon (Hendrick and Hartl, 1993; Hanawa et al., 2000; Hartl and Hayer-Hartl, 2002). Class III heat-shock genes encode for ATP-dependent proteases (ClpC, ClpP, and ClpE) required for degradation of misfolded proteins under stress conditions including high temperature (Figure 1Bb). These proteases are negatively regulated by the CtsR repressor (Figure 1Ab), which is the product of the first gene of the *clpC* operon (Nair et al., 2000). The ClpL protease was recently found to play a considerable role in the elevated temperature tolerance of *L. monocytogenes* AT3E. This finding was observed upon curing the strain from plasmid pLM58, which harbors *clpL*, resulting in a strain with reduced heat resistance. Moreover, insertion of *clpL* increased the resistance of a heat-sensitive *L. monocytogenes* strain (Pöntinen et al., 2017).

#### Resistance to Low Temperatures

*L. monocytogenes* is considered a psychrotolerant bacterium due to its ability to grow at temperatures as low as −0.4°C (Chan and Wiedmann, 2009). This tolerance to cold stress is responsible for the frequent detection of *L. monocytogenes* in refrigerated food products, especially meat, poultry, and seafood (Tasara and Stephan, 2006). Low temperatures result in decreased metabolic rates and changes in membrane composition, expression of cold shock proteins (Csps), and uptake of cryoprotective compounds (Phadtare et al., 1999; Neunlist et al., 2005; Cordero et al., 2016).

The alterations in the membrane in response to cold stress comprise a reduced chain length of fatty acids, an increase in the concentration of unsaturated fatty acids, and altered ratios of *iso*- and *anteiso*-branched fatty acids (Püttman et al., 1993; Russell et al., 1995; Neunlist et al., 2005). These changes maintain fluidity of the membrane at low temperatures and prevent formation of a gel-like state that may result in leakage of cytoplasmic content (Beales, 2004).

Csps are small proteins (65–70 amino acids long) with a highly conserved structure. They bind to single-stranded nucleic acid molecules *via* their ribonucleoprotein binding motifs RNP1 and RNP2 (Horn et al., 2007). This stabilizes the conformation of the nucleic acid and prevents degradation (Barria et al., 2013). Thus, Csps act as molecular chaperones that facilitate replication, transcription, and translation at low temperatures (Lee et al., 2012). CspA, CspB, and CspD contribute to resistance to low temperatures albeit with different importance (Schmid et al., 2009). Interestingly, they also seem to be involved in the resistance to osmotic stress (Schmid et al., 2009). The ferritin-like protein (Flp) was highly induced in response to cold shock suggesting it is involved in response to cold stress (Hébraud and Guzzo, 2000). Chan et al. (2007) determined the cold shock regulon by genome-wide expression analysis and could show that expresion of 105 and 170 genes was increased during growth on 4°C vs. 37°C in logarithmic- and stationary-phase with an overlap of 30 genes including *cspL*. Of these 30 genes, many are involved in membrane and cell wall function, lipid metabolism, transcription or translation.

Another mechanism of *L. monocytogenes* to counteract cold stress is the import of osmolytes such as glycine betaine, carnitine, γ-butyrobetaine, proline betaine, and 3-dimethylsulphoniopropionate as cryoprotectants. In the above mentioned genome-wide transcriptional analysis, the *opuCABCD* operon, which encodes a carnitine transporter, and *gbuC* encoding the substrate binding protein of a glycine betaine transporter showed increased expression in exponential growth phase at 4°C compared to 37°C (Chan et al., 2007). Similarly, expression of *opuCA* and *betL* were increased after exposure of *L. monocytogenes* S1 to cold and freezing stress as shown by quantitative RT-PCR (Miladi et al., 2016). This confirmed previous observations showing that Gbu-mediated betaine uptake improves growth under cold stress and uptake of betaine via BetL and OpuC transport betaine slightly improves cryotolerance (Angelidis and Smith, 2003). In the same study, OpuC was shown to be the main carnitine transporter, which provided markedly higher resistance to cold stress than betaine uptake.

### Resistance to Acidity

Acidification is a method of food preservation widely applied to dairy, meat and vegetable products for centuries and is primarily achieved by fermentation by bacteria either present in the raw food or added as starter cultures (Hill et al., 2017). The preserving effect is achieved, on the one hand, by the metabolic end products, which are weak organic acids (e.g., acetate, lactate) that have anti-microbial activity, and, on the other hand, by inhibition of microbial growth at low pH (Caplice and Fitzgerald, 1999).

Both planktonic and surface attached cells of *L. monocytogenes* display adaptive acid tolerance response (ATR), i.e., bacteria pre-exposed to mild acid stress (pH 5.0) showed higher survival to subsequent challenge at a lower pH (3.0) compared to untreated bacteria (Davis et al., 1996; Chorianopoulos et al., 2011). The extent of ATR may be influenced by the structural properties of the food matrices. For example, *L. monocytogenes* grown on the surface of meat product slices formulated with potassium lactate and sodium diacetate exhibited higher resistance to a pH of 1.5 than the same bacteria exposed to the same pH in homogenates of the meat product (Skandamis et al., 2012). Similar observations were made for *L. monocytogenes* incubated on tomato, lettuce or in culture media for 5 days at 5°C. Bacteria incubated on vegetables were more tolerant to exposure to acidic conditions induced by lactic acid, acetic acid or hydrochloric acid than those kept in tryptic soy broth under the same conditions (Poimenidou et al., 2016).

*L. monocytogenes* has several mechanisms to maintain its internal pH (pH_i_) under acid stress (Table 1) including the F_0_F_1_-ATPase (Cotter et al., 2000), the glutamatic acid decarboxylase (GAD; Feehily et al., 2014), and the arginine and agmatine deiminases (ADI and AgDI; Lund et al., 2014). The F_0_F_1_-ATPase is involved in ATR initiation during mild pH stress (Mclaughlin and Rees, 2009). The GAD system confers resistance to more severe acidic conditions (pH < 4.5; Karatzas et al., 2012) and has also been shown to be activated as result of reduced oxygen availability associated with food atmosphere packaging (Francis et al., 2007; Sewell et al., 2015). It is comprised of two proteins, a cytoplasmic glutamate decarboxylase (GadA or GadB) and a glutamate/GABA antiporter (GadC) located in the cytoplasmic membrane (Cotter et al., 2005). The role of the GAD system is to increase pH_i_ by converting extracellular glutamate to Γ-aminobutyrate (GABA) in an enzymatic reaction that reduces the intracellular proton concentration (Cotter et al., 2001). The ADI and AgDI systems are both involved in the response of *L. monocytogenes* to extreme acidity (Ryan et al., 2009; Soares and Knuckley, 2016). ADI imports arginine molecules from the extracellular environment, converting them to ornithine, CO_2_, ammonia (NH_3_), and ATP. NH_3_ is then protonated to ammonium (NH_4_), which increases pH_i_ (Cotter and Hill, 2003). The same is true for AgDI, which converts agmatine into putrescine and NH_3_ (Chen et al., 2011).

**Table 1:** Genes involved in the acidity resistance of *L. monocytogenes* (data retrieved from two databases, The Universal Protein Resource (UniProt) and The National Center of Biotechnology Information (NCBI), respectively).

Response mechanisms	Genes involved in the response mechanisms	Encoded proteins/enzymes	Class of proteins/enzymes	Location of proteins/enzymes
F_0_F_1_-ATPase	*atpA2* (*lmo2531*)	ATP synthase F1 sector, subunit alfa 2	EC 3.6.3.14 Hydrolase H( + )-transporting two-sector ATPase	Plasma membrane Proton-transporting ATP synthase complex, catalytic core F(1)
	*atpB* (*lmo2535*)	ATP synthase F0 sector, subunit alfa		Integral component of membrane Plasma membrane Proton-transporting ATP synthase complex, coupling factor F(o)
	*atpC* (*lmo2528*)	ATP synthase F1 sector, epsilon subunit		Plasma membrane ATP synthase complex, catalytic core F(1)
	*atpD2 (lmo2529*)	ATP synthase F1 sector, beta 2 subunit		Plasma membrane ATP synthase complex, catalytic core F(1)
	*atp*E (*lmo2534*)	ATP synthase F(0) sector, subunit c		Integral component of membrane Plasma membrane ATP synthase complex, coupling factor F(o)
	*atpF* (*lmo2533*)	ATP synthase F(0) sector, subunit b		Integral component of membrane Plasma membrane Proton-transporting ATP synthase complex, coupling factor F(o)
	*atpG* (*lmo253*0)	ATP synthase F1 sector, gamma subunit		Plasma membrane Proton-transporting ATP synthase complex, catalytic core F(1)
	*atpH* (*lmo2532*)	ATP synthase F(1) sector, delta subunit		Integral component of membrane Proton-transporting ATP synthase complex, catalytic core F(1)
Glutamate decarboxylase activity (GAD) system	*gadA* (*lmo0447*)	Glutamate decarboxylase alpha (GAD-alpha)	EC 4.1.1.15 Decarboxylase, lyase	Cytoplasm
	*gadB* (*lmo2363*)	Glutamate decarboxylase beta (GAD-beta)		
	*gadC* (*lmo2362*)	Putative glutamate:gamma-aminobutyrate antiporter		Cell inner membrane; Multi-pass membrane protein
Arginine deiminase (ADI) system	*arcA* (*lmo0043*)	Arginine deiminase	EC 3.5.3.6 Hydrolase	Cytoplasm
	*arcB* (*lmo0036*)	Ornithine carbamoyltransferase	EC 2.1.3.3 Transferase	Cytosol
	*arcC* (*lmo0039*)	Carbamate kinase	EC 2.7.2.2 Phosphotransferases with a carboxy group as acceptor	Cytosol
Agmatine deiminase (AgDI) system	*aguA1* (*lmo0038*)	Agmatine deiminases 1	EC 3.5.3.12 Agmatine iminohydrolase 1	Cytoplasm
	*aguA2* (*lmo0040*)	Putative agmatine deiminase 2		
	*ptcA* (*lmo0036*)	Putrescine carbamoyltransferase	EC 2.1.3.6 Carbamoyltransferase	Cytoplasm
	*aguC* (*lmo0039*)	Carbamate kinase	EC 2.7.2.2 Transferases	Cytosol
	*lmo0037*	Agmatine/ putrescine antiporter associated with agmatine catabolism		Integral component of membrane

### Resistance to Osmotic Stress

Osmotic stress in food is mostly the result of increased concentrations of salts or sugars that are added to improve the sensory properties and as preserving agents to increase the shelf life of seafood, cheese, salami, pickles, jams, or syrups (Burgess et al., 2016). The presence and concentration of these additives determine water activity (Duché et al., 2002b) and affect bacterial cells by challenging the osmotic balance between cytoplasm and extracellular environment (Bae et al., 2012).

**FIGURE 2:**
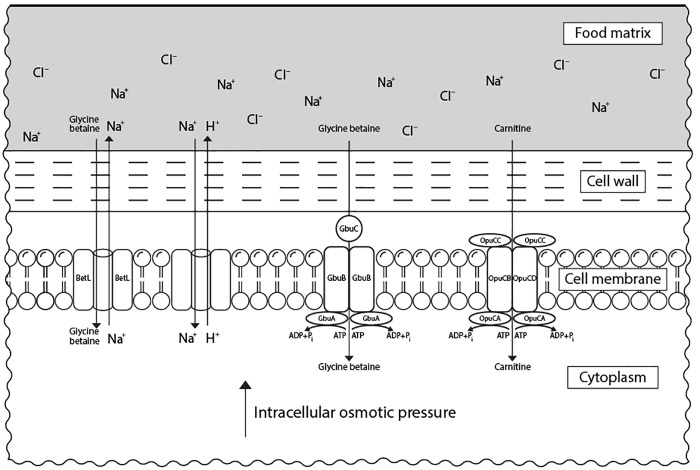
Transport systems of compatible solutes in *L. monocytogenes* associated with its resistance to osmotic stress in food matrices. Under salt stress, *L. monocytogenes* accumulates compatible solutes (carnitine and glycine betaine) *via* specific transport systems from the external medium (food matrix). Carnitine is transported via OpuCABCD system, while glycine betaine can be accumulated via both GbuABC and BetL systems. The presence of compatible solutes in cytoplasm leads to an increase in the intracellular osmotic pressure which restores cell turgor.

In response to elevated concentrations of salt, *L. monocytogenes* accumulates osmolytes, known also as compatible solutes, such as carnitine and glycine betaine in the cytoplasm to reduce osmotic pressure and water loss (Duché et al., 2002a). Besides their property to keep turgor pressure under control, compatible solutes were also shown to stabilize enzymes’ structure and function during stress (Lippert and Galinski, 1992). This mechanism is mediated by an increased expression of genes encoding for proteins involved in the transport of the respective compatible solutes (Cacace et al., 2010; Bae et al., 2012). These are the main carnitine transport system encoded by *opuCABCD* operon, the glycine betaine porter II system, encoded by *gbuABC*, and the sodium-motive-force-dependent glycine betaine uptake system, encoded by *betL* (Chan et al., 2007; Figure 2). L-carnitine is present in raw meat in relevant quantities (Vermassen et al., 2016), while glycine betaine is found in vegetables (e.g., sugar beet, spinach, cereals) (Sleator et al., 1999). OpuCABCD couples ATP hydrolysis to osmolyte transport across the cytoplasmic membrane (Wemekamp-Kamphuis et al., 2004a). This system is formed of OpuCA that hydrolyses ATP providing the energy for transport of the substrate by a complex consisting of the two transmembrane proteins OpuCB and OpuCD and a solute-binding protein OpuCC (Fraser et al., 2000). While BetL is involved in the primary response of *L. monocytogenes* to salt, GbuABC seems to administer the capacity of this bacterium to tolerate such stress during a long-term exposure (Sleator et al., 2003b).

In response to osmotic stress, *L. monocytogenes* can also adjust expression levels of genes other than those associated with osmolytes accumulation. For example, growth of *L. monocytogenes* under salt stress resulted in increased expression of genes for Csps, especially *cspA* and *cspD*, promoting also adaptation to cold stress. The chaperone activity of these proteins is thought to facilitate the repair of DNA lesions, since NaCl has been shown to induce DNA breaks (Dmitrieva et al., 2004; Schmid et al., 2009). On the other hand, Bae and colleagues showed that presence of salt in the growth medium led to a decreased expression of genes associated with carbohydrate PTS systems in *L. monocytogenes* including those related to uptake of β-glucoside, galactitol, fructose, and cellobiose. This suggests a possible connection between a significantly lower growth rate and reduced uptake of carbohydrates under osmotic stress (Bae et al., 2012).

While accumulation of compatible solutes plays the main role in *L. monocytogenes*’ survival to hyper-osmotic shock, a potential response of this bacterium to hypo-osmotic conditions may be mediated by mechanosensitive channels. Bacterial mechanosensitive ion channels regulate turgor pressure by assisting efflux of osmolytes (Perozo and Rees, 2003). So far, genes for two putative mechanosensitive channels have been identified in *L. monocytogenes*. The *lmo2064* gene shows significant homology to *mscL* from *E. coli*, which encodes for a large-conductance mechanosensitive channel (MscL). Additionally, lmo1013 is similar to mscS of Streptococcus pneumoniae encoding for a small-conductance mechanosensitive channel (MscS) (Sleator et al., 2003a; Renier et al., 2012). After subjection to osmotic downshock, *L. monocytogenes* cells have been shown to release almost instantaneously betaine and L-carnitine, which may be linked to the activity of these channels (Verheul et al., 1997a).

### Resistance to Bacteriocins

Bacteriocins are antimicrobial peptides produced by a wide range of LAB and are mostly active against Gram-positive bacteria including *L. monocytogenes* (Cotter et al., 2013; Chikindas et al., 2018). Bacteriocins are natural and safe food additives for a wide range of food products including fruits, vegetables, dairy products, and meat, that are either produced *in situ* by LAB used for food fermentation or added exogenously (Silva et al., 2018). Most bacteriocins are highly specific for their target organisms and kill their targets by inhibiting growth, disruption of membrane homeostasis and pore formation (Zhang and Gallo, 2016).

The only bacteriocin approved as preserving additive in food is nisin, which belongs to the class I bacteriocins and has a broad activity against various Gram-positive bacteria (Cleveland et al., 2001). Nisin is widely used in dairy and meat products with the purpose to inhibit the growth of food-borne pathogens including *L. monocytogenes* and *Clostridium botulinum* (Gharsallaoui et al., 2016). The antimicrobial activity of this bacteriocin is mediateded by two mechanisms. Nisin inhibits the cell wall biosynthesis by binding and sequestering lipid II, which is an essential carrier molecule for peptidoglycan building blocks. Moreover, nisin-lipid II complexes form pores in the membrane leading to permeabilization (Wiedemann et al., 2001).

Resistance of *L. monocytogenes* to nisin has been associated with a series of changes in the cytoplasmic membrane composition aiming to prevent the peptide from crossing this barrier. The studies conducted on nisin-resistant (Nis^r^) cells noticed a reduction in the content of phospholipids with particular emphasis on phosphatidylglycerol and diphosphatidylglycerol, major components correlated with the interaction between nisin and membrane (Ming and Daeschel, 1995; Verheul et al., 1997b). In addition, it was indicated an increase in the proportion of straight-chain fatty acids to the detriment of branched-chain fatty acids, changes that result in a less fluid and, in the same time, more rigid cell membrane (Ming and Daeschel, 1993). The alterations in the cell wall of Nis^r^ strains of *L. monocytogenes* have been also investigated. The resistance of Nis^r^ cells to the degradation action of lysozyme and their sensitivity to benzylpenicillin and ampicillin suggested compositional changes that occurred at the level of this cellular component (Crandall and Montville, 1998). One example could be the D-alanine esterification of teichoic acids (Vadyvaloo et al., 2004). However, a recent transcriptomic analysis of *L. monocytogenes* survival cells following the exposure to a high nisin concentration reported the downregulation of *dltA* and *dltB*, implying that D-alanine residues are not involved in the elevated resistance to this bacteriocin. The study also emphasized the expression regulation of two cell-wall associated genes: downregulation of *lmo2714* encoding for a peptidoglycan anchored protein and upregulation of *lmo2522* encoding for a cell wall-binding protein with possible implication in nisin tolerance (Wu et al., 2018).

In *L. monocytogenes*, nisin resistance is directly mediated by VirR (Grubaugh et al., 2018), the response regulator of the VirRS two component system previously described to be involved in resistance to stress (Mandin et al., 2005). However, in the case of nisin an ABC-transporter encoded by *virAB* seems to be responsible in perception of the stressor instead of the VirS receptor histidine kinase (Grubaugh et al., 2018). VirR mediates the resistance to nisin and other stresses of the cell envelop by regulating the *dltABCD* operon (Kang et al., 2015) that is responsible for modification of lipoteichoic acids (Abachin et al., 2002). Other two component systems that were shown to be involved in resistance to nisin are LiaRS and LisRK (Cotter et al., 2002; Collins et al., 2012; Bergholz et al., 2013). Genes/operons and their products regulated by these TCS with a reported role in resistance to nisin are *lmo2229* (Gravesen et al., 2004; Collins et al., 2012), *telA* (Collins et al., 2010b), *mprF* (Thedieck et al., 2006), *anrAB* (Collins et al., 2010a), and *dltABCD* (Abachin et al., 2002). With the exception of *telA*, all these genes have a known role in metabolism/biosynthesis of components of the membrane or cell wall. Similar to e.g., resistance to pH or salt stress, nisin resistance can also be induced by other stresses, e.g., increased salt concentrations (Bergholz et al., 2013).

Recently, a number of class II bacteriocins with activity against *L. monocytogenes* have been isolated and characterized including pediocins, sakacin P, leucocins, enterococin, mesentericin Y105, garvicin, linocin M18, and others (Eppert et al., 1997; Tosukhowong et al., 2012; Perez et al., 2014; Ovchinnikov et al., 2016; Ríos et al., 2018). *L. monocytogenes* and other bacteria are able to develop resistance to bacteriocins. Natural resistance is observed with a frequency of 1–8% depending on the bacteriocin and the *L. monocytogenes* strain tested (Macwana and Muriana, 2012). Consistent with the receptors and mechanisms of action of bacteriocins, resistant strains show altered expression or mutations in certain phosphotransferase systems (PTSs) (Vadyvaloo et al., 2002, 2004; Tymoszewska et al., 2017). For instance, a spontaneous leucocin-resistant mutant of *L. monocytogenes* B73 was lacking a putative IIAB subunit of a mannose PTS (Ramnath et al., 2000). Furthermore, pediocin PA-1-resistant *L. monocytogenes* 412 mutants overexpressed gene fragments associated with a β-glucoside-specific PTS (Gravesen et al., 2000). Similar results of other studies suggest that resistance of *L. monocytogenes* to class IIa bacteriocins is correlated with a general mechanism consisting of a lack in EII subunits of mannose PTS and a compensatory upregulation of the β-glucoside PTS genes (Dalet et al., 2001; Gravesen et al., 2002).

## Resistance of *L. monocytogenes* to Stress During Processing and Decontamination Using Alternative Technologies

In recent years, a number of novel technologies are applied by the industry for production and preservation of minimally processed foods and diminish the impact of chemical substances on the environment. Consequently this results in new stress conditions encountered by *L. monocytogenes*.

### Resistance to High Hydrostatic Pressure

High pressure processing (HPP) is a technology used in food preservation as an alternative to thermal treatments, aiming to destroy food spoilage microorganisms and food-borne pathogens (Huang et al., 2014). Depending on the food and spoilage organisms, pressures applied for sterilization are usually between 250 and 700 MPa. Bacterial cells subjected to HPP treatments display morphological and physiological changes that may be reversible depending on pressure and holding time. Primary effects of HPP are an increase in the permeability of the cell membrane, the disruption of the protein structure and function, and, as a consequence, inhibition of the metabolism, replication, and transcription (Huang et al., 2014).

The effect of HPP on survival of *L. monocytogenes* was tested under various settings in different food products including cheese (Tomasula et al., 2014), fruit juice (Alpas and Bozoglu, 2003), jams (Préstamo et al., 1999), whole milk (Hayman et al., 2007), and RTE cooked meat products (Hereu et al., 2012). Overall, the results of these studies indicate that resistance of *L. monocytogenes* to HPP varies depending on the strain. For instance, when pressured with 350 MPa at 20°C, *L. monocytogenes* EGD-e displayed only 1.0 log CFU/mL reduction and was more resistant to this HPP treatment than LO28 strain (1.8 log CFU/mL reduction) and ScottA strain (3.2 log CFU/mL reduction) (Van Boeijen et al., 2008). In addition, the type, composition and matrix of food products have an impact on the resistance of bacteria to HPP. Vitamins, amino acids, and cations (Ca^2+^, Mg^2+^) may have protective effects. For example, Mg^2+^ is known to stabilize ribosome structure and Ca^2+^ strengthens the outer membrane (Niven et al., 1999). Also, elevated salt concentrations in a food product may induce uptake of compatible solutes, which in turn stabilize cells during HPP (Abe, 2007). In line with this, a mutant deficient in synthesis of the compatible solute proline showed increased sensitivity to HPP (Considine et al., 2011)

The effect of HPP on *L. monocytogenes* was investigated on the global transcriptomic level by microarray analysis with subsequent RT-PCR on some target genes (Bowman et al., 2008). This indicated that mRNA levels were reduced globally with increasing intensity and duration of the treatment. Nevertheless, HPP induced expression of genes associated with DNA repair, transcription, translation, cell division, protein secretion, motility, chemotaxis, and membrane and cell wall biosynthesis. On the other hand, reduced expression was observed for genes involved in carbohydrates’ uptake, energy metabolism and virulence. Surprisingly, HPP seemed to reduce expression of the general stress sigma factor SigB and part of the SigB regulon. One of the genes showing highest induction by HPP was *cspL* encoding a cold-shock protein. This suggests that HPP also induces cross-resistance to other stresses. For example, HPP resistance in semi-skimmed milk was higher than in buffer and the resistant isolate was also more resistant to heat, acid, and oxidative stress (Karatzas and Bennik, 2002).

Mutations in CtsR, a class III stress genes repressor (Nair et al., 2000), have been linked to spontaneous resistance of *L. monocytogenes* cells to HPP. Mutants with a stable resistance showed point mutations, insertions or deletions in the *ctsR* gene that negatively affected its activity. This loss in *ctsR* function in HPP resistant variants of *L. monocytogenes* was accompanied by increased expression of *clpB*, *clpC*, *clpE*, and *clpP* (Karatzas et al., 2003; Van Boeijen et al., 2010). Clp proteases have a clear role in degradation of misfolded or damaged proteins preventing their potentially harmful accumulation in bacterial cells (Krüger et al., 2000; Tomoyasu et al., 2001). Since protein denaturation is one of the consequences of HPP treatment (Moreirinha et al., 2016) increased Clp protease activity is in line with increased HPP tolerance in *L. monocytogenes*. However, isolation of resistant mutants that do not display these changes indicates that there may be other unknown mechanisms conferring resistance to HPP (Karatzas et al., 2005). Moreover, Chen et al. (2009) reported that different levels of HPP resistance among *L. monocytogenes* strains are not based on *ctsR* gene mutations.

*L. mononcytogene*s ScottA and a spontaneous HPP resistant isolate of this strain were shown to be more resistant to HPP in stationary compared to exponential growth phase (Karatzas and Bennik, 2002). Moreover, it seems that cells in stationary phase of growth do not exhibit the highest resistance to HPP treatment. *L. monocytogenes* cells found in long-term-survival phase showed even higher HPP tolerance, as transition back to log and stationary phases resulted in less survivors after pressurization. This phenomenon has been attributed to a change in cell morphology from rods to cocci that results in cytoplasmic condensation and, implicitly, reduction of intracellular water activity (Wen et al., 2009).

### Resistance to UV-Light

Another more recent method of food decontamination, included under the umbrella of alternative technologies, is pulsed or continuous UV-light, which kills microorganisms found on the surface of food products as result of cross-contamination occurring during processing procedures such as cutting, slicing or packing (Gómez-López et al., 2007). Although approved by the United States and Food and Drug Administration (USFDA) for food application in 1996, the safety of this technology regarding the potential of permanent microbial inactivation still remains under question.

The bactericidal effect of UV light is caused by DNA damage as a consequence of the formation of photoproducts including cyclobutane-pyrimidine dimers (CPDs), pyrimidine 6-4 pyrimidone photoproducts (6-4PPs), and their Dewar isomers (Rastogi et al., 2010). Other mechanisms of bacteria inactivation caused by UV light are the photophysical and photothermal effects resulting in leakage of cellular content following the absorption of the high energy light pulses (Gómez-López et al., 2007). The efficacy of UV treatment in decontamination of food surfaces depends on a number of factors including the food product, distance and position of the product to the light source, energy level given by number and frequency of the light pulses, level of contamination and others (Gómez-López et al., 2007). The potential of UV-C light in *L. monocytogenes* inactivation was shown to be lower on fruits with smooth surface (apples and pears) compared to fruits with a rougher surface (cantaloupe, strawberry or raspberry) (Adhikari et al., 2015). UV light is also used as disinfection procedure to improve hygiene in food processing environments (Bintsis et al., 2000). The presence of organic materials such as food debris on stainless steel surfaces appeared to protect *L. monocytogenes* cells against UV-C radiation (Bernbom et al., 2011).

Several studies have been conducted in order to investigate the efficacy of *L. monocytogenes* inactivation by pulsed UV-light on/within various food matrices. A maximum of inactivation of *L. monocytogenes* ScottA on the skin side of salmon filets was achieved with 180 pulses of UV light of 5.6 J/cm^2^ at a distance of 8 cm for 60 s and efficacy was markedly lower on the muscle side (Ozer and Demirci, 2006). Similarly, the best inactivation rates of the same strain on chicken frankfurters was obtained with 180 pulses in 60 s at UV energy of 1.27/cm^2^ (Keklik et al., 2009).

*L. monocytogenes* has been reported to be more resistant to UV-light than other pathogens, such as *E. coli* (Beauchamp and Lacroix, 2012). However, very little is known regarding specific mechanisms of UV resistance in *L. monocytogenes*. Sublethal challenge with other stresses does not induce cross-resistance to UV light and UV resistance does not seem to depend on SigB, the general stress sigma factor of *L. monocytogenes* (Gayán et al., 2015). Global gene expression analysis of the response to both pulsed light (PL) and continuous ultraviolet treatment was conducted in *L. monocytogenes* 10403S (Uesugi et al., 2016). Although the overall amplitude of the changes in gene expression was low, a number of genes encoding for stress proteins, motility and transcriptional regulators were induced by UV exposure. However, no increased expression was observed for *lmo0588*. This gene encodes for a (putative) photolyase. This protein plays an important role in photoreactivation, which is the recovery of bacteria sublethally injured by UV light due to subsequent exposure of visible light (Gómez-López et al., 2007). During photoreactivation, photolyase binds and repairs the pyrimidine DNA lesions using light energy absorbed by its chromophores (Sinha and Häder, 2002). In fact, an increase in viability was observed for a UV-treated *L. monocytogenes* serotype 1/2b after incubation in daylight for only 90 min followed by storage under dark (Lasagabaster and Martínez de Marañón, 2017).

### Resistance to Pulsed Electric Fields

Pulsed electric fields (PEF) processing is another non-thermal alternative technology for decontamination mainly used in liquid foods processing and thus is not limited to inactivation on the surface of a product. The treatment consists of short, highly intense pulses of electric fields applied to the products in order to achieve the inactivation of unwanted microorganisms (Góngora-Nieto et al., 2002). The inactivating effects of PEF are destabilization and, depending on the strength of the PEF, irreversible damage of the cytoplasmic membrane with formation of micropores and leakage of cytoplasmic content (Góngora-Nieto et al., 2002). Similar to HPP and UV light but unlike conventional thermal food processing technologies, such as pasteurization, this method is less detrimental to food matrices and better in preserving the sensory and nutritional characteristics of the product (Toepfl et al., 2007). The efficacy of inactivation by PEF is determined by a number of factors related to the process (strength, duration, frequency of the pulses, temperature, etc.), the food product (composition, conductivity, pH, etc.) and the microorganisms to be inactivated (species, growth phase, etc.) (Wouters et al., 2001).

In general, Gram-positive organisms are believed to be more resistant to PEF than Gram-negative bacteria, presumably due to the thicker cell wall and stiffening (lipo)teichoic acids (Lado and Yousef, 2002). For example, *L. monocytogenes* proved to be more PEF tolerant than *Salmonella enteritidis* and *E. coli* when treated in melon and watermelon juices (Mosqueda-Melar et al., 2007). Thus, PEF alone is probably not the method of choice for inactivation of *L. monocytogenes*. It has been recommended to combine PEF with other methods such as ozone (Unal et al., 2001), mild heat (Fleischman et al., 2004) or plants infusions with antimicrobial properties (Rivas et al., 2016) to decontaminate food products at risk for contamination with *L. monocytogenes*. Low inactivation rates were observed for *L. monocytogenes* in a Spanish vegetable-based beverage and this was attributed to the neutral pH of the product (Selma et al., 2006). In fact, in buffer inactivation rates of *L. monocytogenes* by PFE were higher at acidic pH (Álvarez et al., 2002; Gómez et al., 2005; Saldaña et al., 2009). Further data in buffered systems or culture media indicated that resistance to PEF was increased in stationary growth phase and in media with reduced water activities (Álvarez et al., 2002; Lado and Yousef, 2003) suggesting a cross-resistance with other stresses.

Besides membrane disruption, PEF was suggested to affect bacterial cells by denaturation of the membrane-bound proteins as result of localized overheating caused by the capacity of the formed pores to conduct electricity (Simpson et al., 1999). This might imply an involvement of chaperones in the response of *L. monocytogenes* to PEF. One study compared the expression levels of three major molecular chaperones, namely GroEL, GroES, and DnaJ, in a resistant and a sensitive *L. monocytogenes* strain treated with a sublethal PFE challenge and found a transient reduction in expression of these chaperones in the sensitive strain (Lado et al., 2004).

Somolinos and colleagues have shown no difference in the resistance to PEF processing between *L. monocytogenes* EGD-e and its isogenic Δ*sigB* mutant suggesting that SigB is not involved in the repair mechanism of injured cells as shown for thermal treatment of the same strains. Also, unlike heat challenge, mild acid shock applied to *L. monocytogens* cells did not increase the resistance to subsequent PEF treatment (Somolinos et al., 2010).

### Resistance to Oxidative Stress

Under oxidative stress (bacterial) cells encounter high concentrations of oxygen radicals (Suo et al., 2014). This disturbs the normal redox state of cells leading to cell death due to the oxidative damage of proteins, lipids and nucleic acids. Bacteria use reduction pathways that repair damage of susceptible amino acids (cysteine and methionine) induced by reactive oxygen (ROS) or reactive chlorine species (RCS). ROS are a group of compounds containing oxygen on different redox states such as hydrogen peroxide, hydroxyl radical or peroxyl radical. In bacteria, these compounds activate enzymes such as superoxide dismutases (SOD), catalases, peroxidases and efflux pumps to counteract oxidative stress (Dröge, 2003; Archambaud et al., 2006).

Recently, Harter and colleagues revealed the presence of a novel stress survival islet (SSI-2) in *L. monocytogenes* ST121 and other strains isolated from food and food processing environments. The SSI-2 consists of two genes *lin*0464 and *lin*0465 (PfpI protease), that are upregulated after 10 min of exposure to oxidative stress. Lin0464 seems to be a positive gene regulator of *lin0465*, because the time frame of increased transcription of *lin0465* is longer compared to that of *lin0464* and because the constitutive expression of *lin0464* has no effect on the survival rate in Δ*lin0465* mutant. Under alkaline or oxidative stress encountered in food processing environments, the expression of both genes offers *L. monocytogenes* ST121 the possibility to adapt and survive, an independent response mechanism from the alternative sigma factor (Harter et al., 2017).

Even if SigB is the main regulator of stress genes, its role in the oxidative stress resistance is controversial. A number of authors (Ferreira et al., 2001; Oliver et al., 2010) provided experimental data suggesting that, in *L. monocytogenes*, oxidative stress protection is conferred by σ^B^ since Δ*sigB* mutant cells are sensitive to this stress. Other studies suggested that *sigB* expression is harmful for stationary-phase *L.* *monocytogenes* (EGD-e and 10403S) cells grown aerobically, under oxidative stress conditions mediated by hydrogen peroxide. Furthermore, Δ*sigB* mutant proved, besides oxidative stress resistance, a stronger catalase activity upon addition of 30% H_2_O_2_, compared to the wild type. Interestingly, no difference was observed in the transcription of the catalase gene between the Δ*sigB* mutant and the wild type (Boura et al., 2016). All these discrepancies within the role of *sigB* in oxidative stress response may be explained by variation between strains (Moorhead and Dykes, 2003), oxidative agents tested, differences in growth phase, and oxygen tension of the culture (Boura et al., 2016).

In *L. monocytogenes*, the resistance to oxidative stress was also correlated with biofilm formation (Suo et al., 2012). Four genes related to oxidative stress, *kat*, *perR* (peroxide operon regulator), *sigB* and *recA* (recombinase A) were upregulated in a Δ*sod* mutant, which produced more ROS than the wild-type *L. monocytogenes* 4b G (Suo et al., 2014). Also, a Δ*perR* *L. monocytogenes* mutant showed increased sensitivity to hydrogen peroxide stress. Moreover, catalase activity in these cells increased to a toxic level resulting in smaller colonies and changes in cell morphology compared to the wild type (Rea et al., 2005).

The anti-oxidative *kat* gene acts synergistically with *sod* gene (superoxide dismutase), both being involved in the protection against toxic effects of hydrogen peroxide and superoxide anion radicals (Suo et al., 2012). The *sodA* gene encodes for MnSOD, a cytosolic SOD enzyme which uses manganese in the catalytic reactions. Archambaud and colleagues reported that, during stationary phase, *L. monocytogenes* MnSOD activity is downregulated by phosphorylation at serine/threonine residues. MnSOD activity increases only when dephosphorylation is performed, condition that facilitates its secretion in the bacterial culture media via SecA2 pathway (Archambaud et al., 2006).

Other genes involved in the response to oxidative stress are *fri*, *gltB*, and *gltC*. Based on its iron-binding activity, *fri*-encoded ferritin detoxifies oxidative agents (Dussurget et al., 2005; Olsen et al., 2005). Huang and colleagues introduced a role of *glt*B and *glt*C gene products in oxidative stress and *L. monocytogenes* biofilm formation. GltC is a member of LysR-type transcriptional regulator and *gltB* encodes for a glutamate synthase regulated by GltC. Experiments with *gltB* and *gltC* mutants revealed a reduced ability to form biofilm and an increased sensitivity to oxidative stress (Huang et al., 2013).

## Conclusion

*L. monocytogenes* is able to use diverse mechanisms to survive various stress conditions encountered in food matrices. This explains the efforts made by scientists to understand these mechanisms in order to develop more efficient methods to reduce *L. monocytogenes* occurrence in food and food related environments. With the present review, we aim at providing an overview of the current knowledge on food-related stress and stress resistance of *L. monocytogenes*. As observed for many other organisms, *L. monocytogenes* employs different survival mechanisms for the same stress or use the same mechanism for different stresses (heat-shock genes are expressed when *L. monocytogenes* is subjected to heat stress, HPP or PEF; cold-shock genes are expressed and osmolytes transport systems are activated when *L. monocytogenes* encounters cold or osmotic stress). However, compared to other organisms, the large number of mechanisms also increases the possibilities of this organism for cross-resistance.

The ongoing trend toward healthier, minimally processed food products with unaltered sensory and nutritional properties demands new strategies for food preservation, while no compromises are accepted for food safety. Alternative treatments (e.g., high pressure, pulsed electrical field, UV light), have yielded promising results, but their application often allows to *L. monocytogenes* recovery. However, current data also suggest that combinations of these techniques with e.g., natural preserving additives such as bacteriocins may be feasible solutions. Nevertheless, the effects on and resistance of *L. monocytogenes* to such combinations of stresses need to be investigated.

## Author Contributions

FB and LG-G drafted the manuscript and all authors revised and contributed to its final version.

## Conflict of Interest Statement

The authors declare that the research was conducted in the absence of any commercial or financial relationships that could be construed as a potential conflict of interest.

## Footnotes

1 https://www.theguardian.com/business/2018/jul/06/uk-supermarkets-recall-frozen-vegetables-over-bacteria-fears (Accessed 31.07.2018)

2 https://www.fda.gov/AJAX/All/ (Accessed 31.07.2018)

3 http://www.who.int/csr/don/02-may-2018-listeriosis-south-africa/en/ (Accessed 10.10.2018)
